# Experimental Study on the Energy-Release Characteristics of Fine-Grained Fe/Al Energetic Jets under Impact Loading

**DOI:** 10.3390/ma12203317

**Published:** 2019-10-11

**Authors:** Qiang Li, Ye Du

**Affiliations:** College of Mechatronic Engineering, North University of China, Taiyuan 030051, China; dy_ibc@nuc.edu.cn

**Keywords:** fine-grained Fe/Al, energetic jets, impact loading, impact energy, energy release

## Abstract

The energy released by the active metal phase in fine-grained Fe/Al energetic materials enables the replacement of conventional materials in new types of weapons. This paper describes an experiment designed to study the energy-release characteristics of fine-grained Fe/Al energetic jets under impact loading. By means of dynamic mechanical properties analysis, the physical and chemical properties of Fe/Al energetic materials with specific content are studied, and the preparation process is determined. The energy-release properties of fine-grained Fe/Al jets subject to different impact conditions are studied based on experimental data, and energy-release differences are discussed. The results show that for fine-grained Fe/Al energetic materials to remain active and exhibit high strength, the highest sintering temperature is 550 °C. With increasing impact energy, the energy release of fine-grained Fe/Al energetic jets increases. At an impact-energy threshold of 121.1 J/mm^2^, the chemical reaction of the fine-grained Fe/Al energetic jets is saturated. The experimental data and microscopic analysis show that when the impact energy reaches the threshold, the energy efficiency ratio of Fe/Al energetic jets can reach 95.3%.

## 1. Introduction

The energy released by the active metal phase in energetic materials enables the replacement of conventional materials in new types of weapons [1,2,3]. Researchers have found that grain refining can greatly increase the strength and reactivity of Fe/Al composites [4,5,6]. If a proper preparation process is used, the compressive strength of Fe/Al composites can be significantly improved while maintaining the chemical reactivity. Generally, Fe/Al composites have high strength, are inert after sinter hardening, and are insensitive to friction, combustion, and explosion under normal conditions. However, under a strong impact load, the impact energy drives the iron phase and a large amount of the active aluminum phase in the sintered material to react violently, releasing a large amount of energy; thus, these composites can replace conventional inert materials in weaponry, such as fragmentations and liners used to form jets, efficiently damaging the target.

Recently, substantial progress was made in the field of Fe/Al composite materials research. Airiskallio et al. [7] found that the intermetallic compounds formed from Fe/Al composites have excellent oxidation resistance. Wang et al. [8] prepared Fe/Al micro/nanocomposite particles with core-shell structures and found that the thermal reactivity of the Fe/Al micro/nanocomposite powder was significantly higher than that of the raw Al powder by differential scanning calorimetry (DSC) analysis. Wang et al. [9] studied the effect of the composition ratio on the reaction heat of Fe/Al energetic materials by means of thermal analysis and micro-characterization and selected the most active Fe/Al ratio.

The above reports mainly focused on the physicochemical properties of static Fe/Al energetic materials by means of thermal and microstructural analyzes. However, experimental studies on the impact-energy-release characteristics of Fe/Al energetic materials under high strain rates and high impact loading are still insufficient, and there is no research on applying Fe/Al energetic materials to jets. In this study, traditional micron-sized aluminum and iron particles were refined and modified by high-energy ball milling. By optimizing the preparation process, the material can be guaranteed to have high strength while maintaining its activity when it is prepared as a liner. X-ray diffraction (XRD), differential scanning calorimetry (DSC), and scanning electron microscopy (SEM) techniques are used to analyze the compositions and morphologies of the intermetallic composites formed under different preparation conditions, and the physical and chemical trends of Fe/Al energetic materials are obtained. The dynamic compressive mechanical properties of fine-grained Fe/Al energetic materials are studied using a split Hopkinson pressure bar (SHPB) system. Introducing the concept of impact energy, an experiment is designed to study the reaction characteristics of fine-grained Fe/Al energetic jets under different impact energies, and the differences in energy release are discussed.

## 2. Materials and Methods

### 2.1. Preparation of Experimental Samples

A mixture of fine-grained Fe and Al is a typical energetic structural material. Reactive Al and Fe in Fe/Al composites react rapidly with ambient oxygen under impact loading, according to Equations (1) and (2):

Al + 3/4 O_2_ = 1/2 Al_2_O_3_ (1)

Fe +2/3 O_2_ = 1/3 Fe_3_O_4_ (2)

This study considers Fe/Al composites with mass ratios of 40/60, which were determined according to stoichiometry and the related literature [10]. The particle sizes of the Al and Fe (purchased from Beijing Xing Rong Yuan Technology Co., Ltd.) powders were 3.8 × 10^3^ nm and 3.1 × 10^3^ nm, respectively. The Al and Fe powders were mixed and milled to fine grain in a ball mill (QM-2, Changsha Tianchuang Instrument Factory, high aluminum ceramic balls with 1 and 3 mm, ball to powder mass ratio of 10:1). The rotational speed was 200 r/min, and the milling time was 6 h. The parameters for the Fe/Al composites are shown in Table 1.

**Figure 1:**
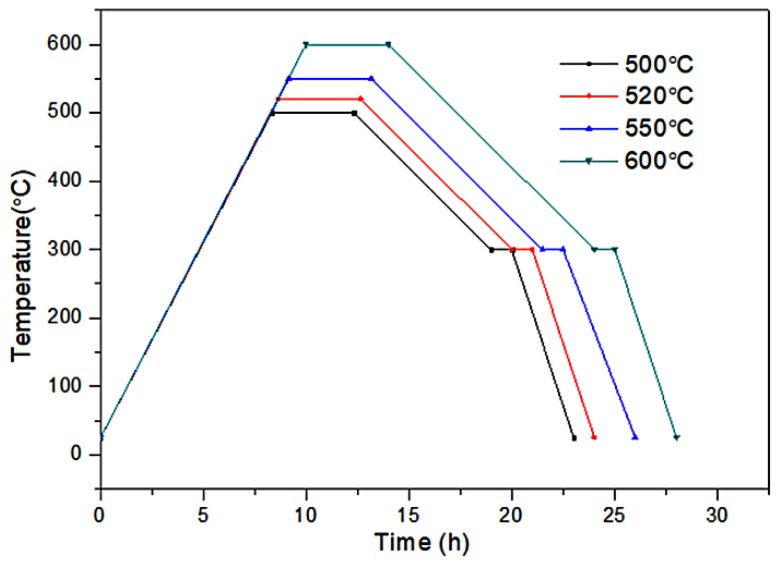
The temperature steps of the sintering cycles.

**Figure 2:**
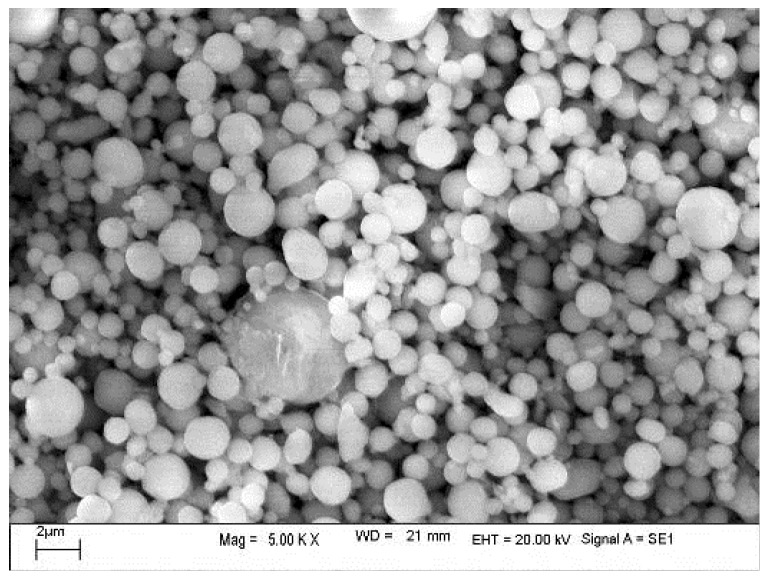
SEM image of the Al powder.

**Figure 3:**
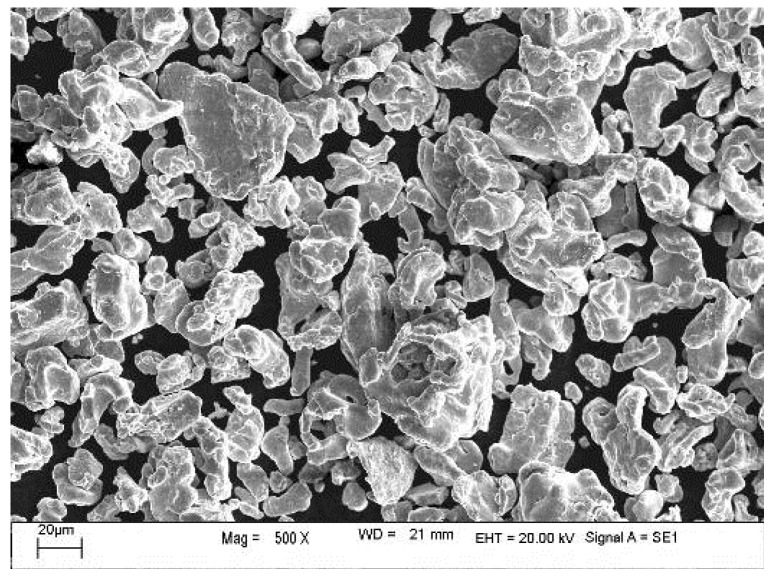
SEM image of the Fe powder.

To obtain a high strength while maintaining activity, the fine-grained Fe/Al composites were prepared as follows:

(1) The mixed powders were loaded into a custom mold and sintered in a vacuum hot-pressing sintering furnace (R-C-ZKQY-07, Chenrong Electric Furnace Co., Ltd. Shanghai, China), and high-purity nitrogen was used as a protective gas.
(2) After 15 min, the power supply for the heater was turned on, and the water-cooling system was turned on when the temperature reached 150 °C. The heating rate was set to 1 °C/min. The sintering pressure was maintained at 10 MPa.
(3) After the temperature reached the highest sintering temperature (500, 520, 550, or 600 °C), the temperature was maintained for 4 h; then, the mould was cooled to 300 °C at a cooling rate of 30 °C/h. This temperature was maintained for 1 h.
(4) As the temperature was gradually lowered, most of the pressure was released. After the temperature reached room temperature, the mold was removed. The preparation process for the Fe/Al composites is shown in Figure 1. SEM images of the Al and Fe powders are shown in Figure 2 and Figure 3, respectively.

### 2.2. Microstructure Analysis of the Composites

**Figure 4:**
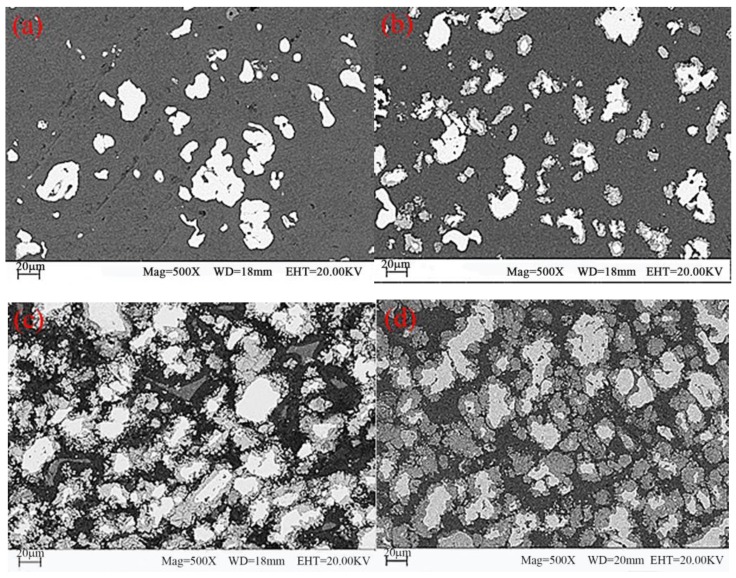
SEM images of the Fe/Al composites. (**a**) 500 °C, (**b**) 520 °C, (**c**) 550°C, and (**d**) 600 °C.

**Figure 5:**
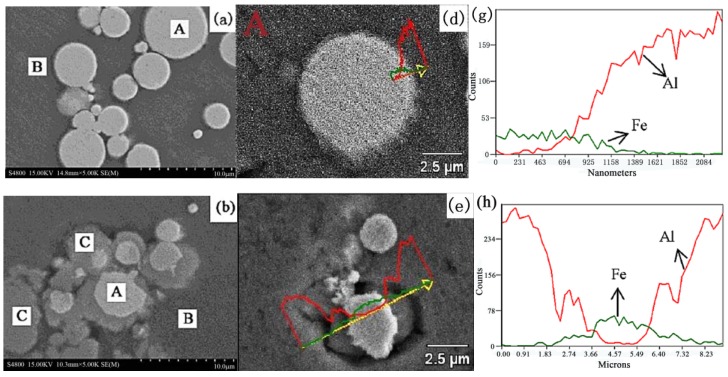

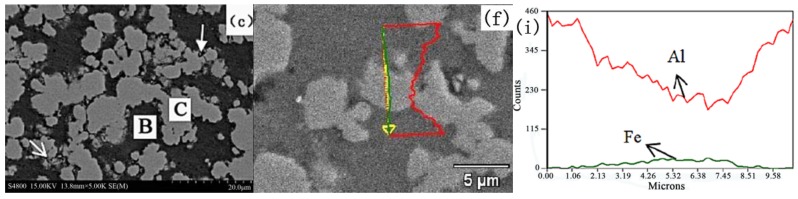
DSC tests of the Fe/Al composites with different maximum sintering temperatures. (**a**,**d**,**g**) 520 °C, (**b**,**e**,**h**) 550 °C, and (**c**,**f**,**i**) 600 °C.

**Figure 6:**
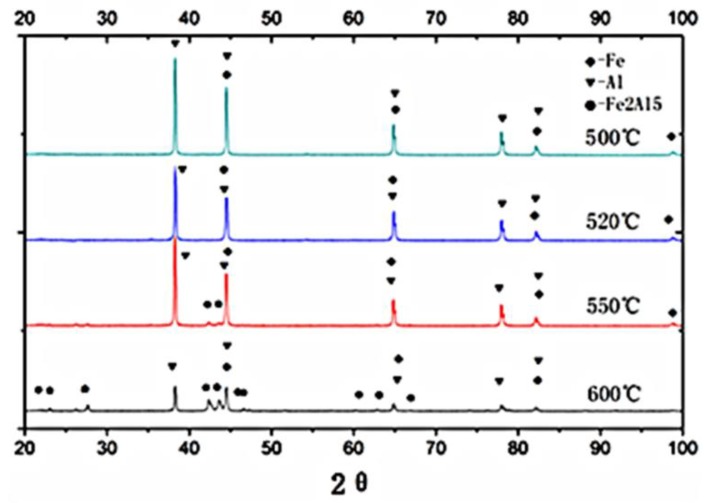
XRD patterns of the Fe/Al composites with different maximum sintering temperatures.

The microstructure of the Fe/Al composites was determined using SEM (S-4800, Hitachi Corporation, Japan), as shown in Figure 4. Figure 5 shows the DSC (DSC404F3, Netzsch, Germany) analysis of fine-grained Fe/Al composites sintered at different temperatures. (a–c are the local sampling graphs of Figure 4. d–f are the energy-dispersive line-scanning paths of the corresponding regions of a–c, respectively. Images g–i are the results of the energy-dispersive scanning analysis.) The fine-grained Fe/Al composites were subjected to XRD (D8advance, Bruker Optics, Germany) analysis, as shown in Figure 6.

Figure 4, Figure 5 and Figure 6 show that when the maximum sintering temperature is 500 °C and 520 °C, most of the composites have only an iron phase and aluminum phase, corresponding to area A and area B in Figure 5, respectively. These phases do not undergo a chemical reaction during the preparation process, and the iron phase is coated by the aluminum phase. When the maximum sintering temperature is increased to 550 °C, a weak Fe_2_Al_5_ diffraction peak appears in the XRD pattern, indicating that a small amount of Fe_2_Al_5_ is present, corresponding to area C in Figure 5. Since the temperature has not reached the melting point of aluminum or the eutectic point of aluminum–iron, a solid-state diffusion reaction between Al and Fe occurs at this time, as shown in Equation (3):

2Fe + 5Al = Fe_2_Al_5_ (3)

When the temperature is further increased to 600 °C, the intensity of the diffraction peak of the Fe_2_Al_5_ phase remarkably increases, indicating that a considerable amount of Fe_2_Al_5_ is present. At the same time, the iron phase and the aluminum phase rapidly decrease, indicating that a diffusion reaction occurred at this temperature. No new substances are detected except for Fe_2_Al_5_ during the entire heating process because the synthetic rate of Fe_2_Al_5_ is much higher than that of FeAl_2_, FeAl_3_ and other aluminum-rich phases [10,11] under normal-pressure or low-pressure conditions and sintering at temperatures below 600 °C. FeAl_2_, FeAl_3_, and other aluminum-rich phases are rarely detected by XRD.

According to the above analysis, to maintain the activity of the composites, it is necessary to retain sufficient aluminum and iron phases during sintering, and the maximum sintering temperature should be maintained below 550 °C.

## 3. Experimental Design and Scheme

### 3.1. Dynamic Compression Properties Experiment

**Figure 7:**
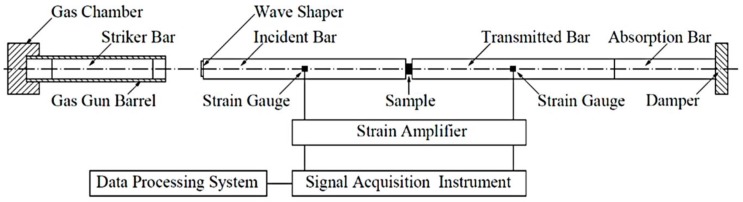
Schematic diagram of the SHPB test system.

The dynamic compressive mechanical properties of Fe (40)/Al (60) composites sintered at 500, 520, 550, and 600 °C were tested on a SHPB system (Key Laboratory of Science and Technology on Materials under Shock and Impact, Beijing, China) at a strain rate of 5500 s^−1^. The experimental system consisted of a gas gun, a striker bar, an incident bar, a transmission bar, an absorption bar, and a data acquisition system. Different strain rates were obtained by changing the atmospheric pressure applied to the impact rod [12]. The incident wave, reflected wave, and transmitted wave were recorded on a digital oscilloscope by means of the strain gauge and preamplifier attached to the incident rod and transmission rod, respectively. The stress–strain relationship of the material was obtained by processing the original data. The SHPB test system is shown in Figure 7.

### 3.2. Experiment on the Energy-Release Characteristics under Impact Loading

Due to the limitations of the experimental conditions, it is impossible to directly measure the energy released by the Al/Fe energetic jet under impact loading. Therefore, this experiment was designed to collect the pressure signal from the energy released by the fine-grained Fe/Al energetic jet in an enclosed space and convert the overpressure signal into an energy value through a certain calculation method. First, the fine-grained Fe/Al liner forms an energetic jet driven by charge. After impacting the target, the Fe/Al energetic jet reacts chemically and releases energy. The air in the quasi-hermetic container is compressed to create pressure. The sensor on the wall of the container receives the pressure signal and converts it into an electric signal. The energy-release characteristics of the Fe/Al energetic jet are obtained by data processing and analysis. To characterize the impact loading on the jet, the concept of "impact energy" is introduced. By adjusting the thickness of the target plate, the jet is subjected to different impact energies when impacting the target.

**Figure 8:**
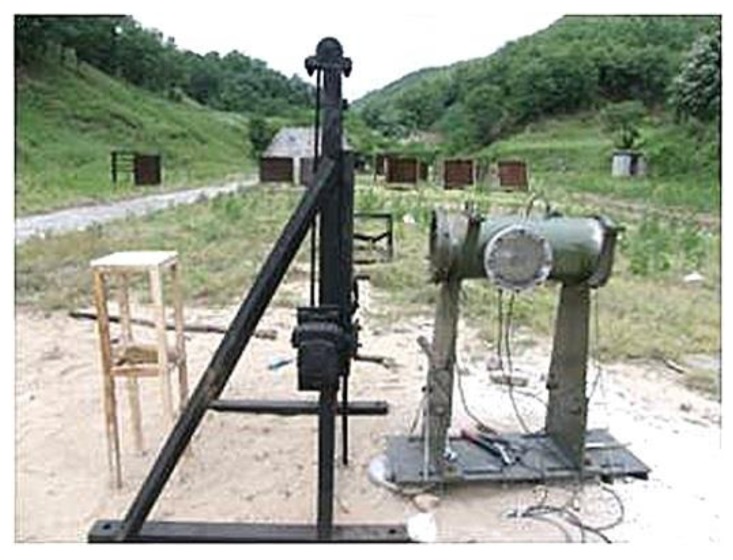
Field experimental device.

**Figure 9:**
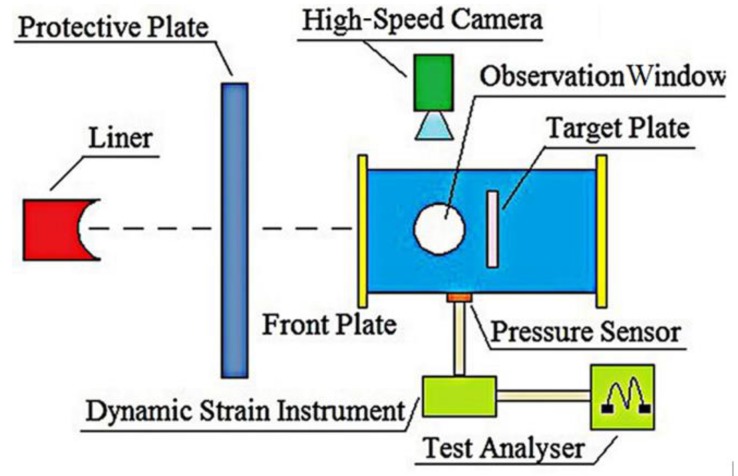
Schematic diagram of the energy-acquisition system.

The impact-induced reaction experimental system consisted of a Fe/Al energetic liner (with shape of hemisphere, Φ40, with equal wall thickness of 2 mm, with equivalent cone angle of 120°), a charge (poly-8, density: 1787 kg/m^3^, detonation velocity: 8390 m/s), a Φ380 (mm) steel test container (volume: 14.67 L), a pressure-testing system (BZ2202 multi-channel dynamic strain gauge, TST3125 dynamic test analyzer), a pressure sensor (range: 5 V, sampling rate: 20 kHz, sampling length: 58 s, delay: 2 ms, control level: 0.15 V), several wires, front and rear sealing plates, several Q235 steel target plates with different thicknesses, a steel protective plate, a high-speed camera, and several support brackets. The field experimental device is shown in Figure 8, and the experimental system is shown in Figure 9. The target plate was fixed to the steel support bracket inside the test container via bolts, and a protective plate was placed between the test vessel and the liner to prevent detonation of the products and minimize the impact on the test system. To ensure that the jet entered the test vessel smoothly, 80 mm pressure-relief holes were placed in the center of the protective plate and the front sealing plate. The axis of the liner was calibrated with a laser, and the heights of the protective plate and the front sealing plate were adjusted so that they were the same as that of the axis of the liner. The liner was placed on the launcher and was driven by charge action to form a jet, which passed through the pre-perforated protective plate and impacted the steel baffle to produce an intense chemical reaction, thus releasing a large amount of heat and causing the air in the chamber to expand and produce overpressure conditions. A sensor on the wall of the test container recorded the voltage–time signal due to the energy released by the energetic material in the jet, and the original signal was smoothed after filtering. The energy-release pressure–time curve was obtained by numerical calculation. High-speed photography was used to observe the macroscopic energy-release phenomenon, and data analysis of the pressure–time curve was carried out. An XRD analysis method was used to observe and test the composition of the residual substance in the container.

The mass ratio of Fe/Al was 4:6, and the maximum sintering temperature of the liner was 550 °C. The thickness of the target plate was 2–5 mm to investigate the effect of different impact energies on the release characteristics of the Fe/Al energetic jet. To compare the impact-energy-release effect of the Fe/Al energetic jet and inert jet, pure inert copper and an Fe/Al mixture prepared without hot-pressing sintering were added as a control group. Three experiments were carried out for each group, and the results were averaged. The experimental scheme is shown in Table 2.

## 4. Results and Discussion

### 4.1. Dynamic Compression Properties

**Figure 10:**
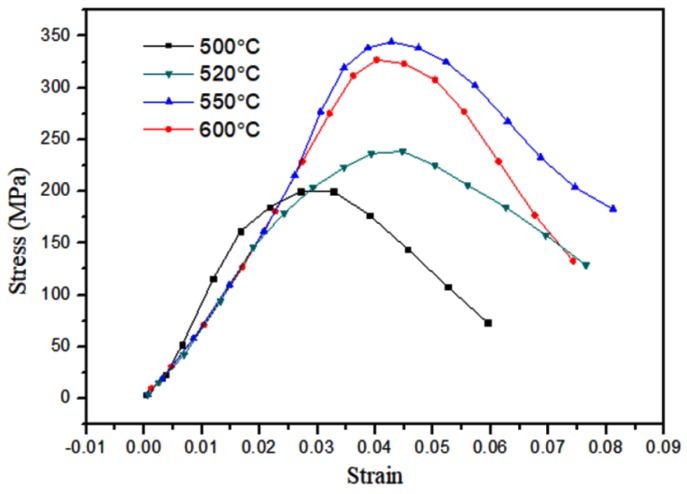
Dynamic compression curves of the Fe/Al composites with different maximum sintering temperatures at a strain rate of 5500 s^−1^.

**Figure 11:**
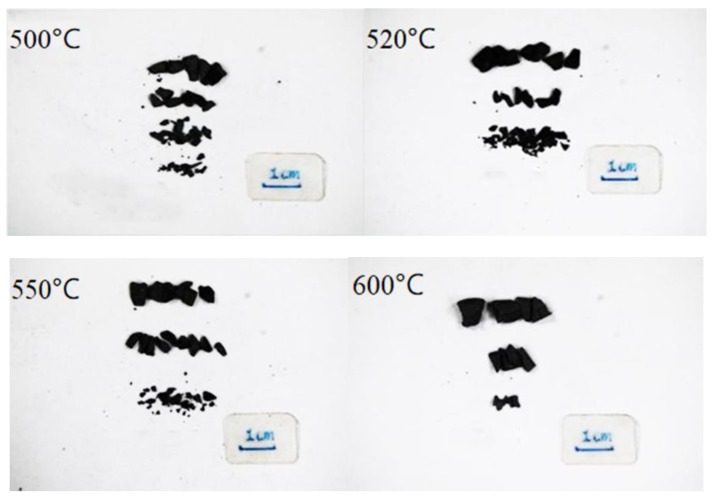
Images of the compression fragments.

**Figure 12:**
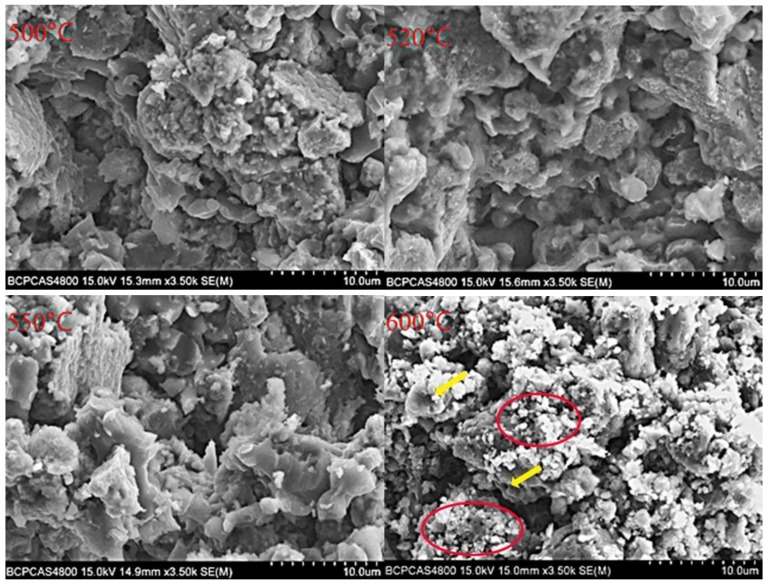
SEM micrographs of the fractured composites.

Figure 10 shows the dynamic compression curves of the Fe (40)/Al (60) composites sintered at different maximum sintering temperatures. Figure 11 shows photographs of the resulting fragments after dynamic compression. Figure 12 shows the SEM topography of the fragments of dynamically compressed composites.

In Figure 10, the stress–strain curves of the four different sintering temperatures at a strain rate of 5500 s^−1^ do not show the yielding platform or yielding peaks common to plastic metals. After the composites reach their compressive strengths, they break and unload, reflecting the characteristics of a brittle material. When the maximum sintering temperature is 550 °C, the compressive strength of the sample is at its highest. With the increasing sintering temperature, the fracture strain values are 0.031, 0.043, 0.041, and 0.043. The toughness of the material does not change much when the sintering temperature exceeds 520 °C. The strain is less than 5% at all four temperatures, and the ductility of the material is poor. The pictures in Figure 11 show that the composites are broken into several fragments in the stress-concentration area, and the fracture surface is perpendicular to the principal stress, which further proves that brittle fracturing occurred. During the sintering process, intermetallic composites are produced by the solid-state diffusion and chemical reaction of the composites. An uneven distribution of grains results in the existence of intergranular pore defects. These defects are activated by the impact load, and cracks continuously form and propagate. The cracks intersect with each other to form a network fragmentation zone, which is reflected in the macroscopic fragmentation of the composites.

Figure 12 shows that when the sintering temperature is 520, 550, or 580 °C, there are no dimples and fibrous structures on the fractured surfaces of the composites, and the fracture is intergranular brittle fracture. This result is due to the existence of a network fragmentation zone, which results in the surfaces of an intergranular fracture being rougher. A small number of dimples are found in the fracture morphology of the composites sintered at 600 °C, as shown by the yellow arrow, which indicates that the material has a tendency toward ductile fracturing. As the surface roughness of the composites increases, many small white particles are present on the surface, and an obvious spalling phenomenon can be seen in the red circle in the figure. These factors are a result of Al particles diffusing into Fe particles and intermediate products, resulting in pore formation at the original positions of the Al particles, based on the Kirkendall effect, when the Fe/Al composites are sintered at 600 °C. These voids are distributed along the edges of the intermediate products and expand along the grain boundaries of the brittle phase after being subjected to an impact load, forming numerous deep cracks. When the cracks converge at the surface, they cause spalling and brittle fracturing. The fine white particles on the surface are aluminum particles that coat the brittle phase.

According to the results of 2.2 and 4.1, the Fe/Al energetic composite with the sintering temperature of 550 °C has the highest compressive strength and contains enough aluminum and iron phases to maintain its chemical activity. In the subsequent study on the impact energy-release characteristics, the Fe/Al energetic jet created under this preparation process is the research objective.

### 4.2. Impact-Induced Reaction Characteristics

To quantitatively study the effect of impact loading on the energy-release characteristics of fine-grained Fe/Al energetic jets, the concept of “impact energy” is introduced in this paper. According to previous reports, the main mechanism of the chemical reaction of a metal under impact conditions is that the shockwave changes the internal structure and order of the material and causes a certain increase in temperature [13], resulting in a high-temperature and high-pressure environment in which a local hot spot forms and a rapid diffusion reaction occurs. The reaction principle for energetic jets, such as explosives, is basically the same. Therefore, the level of “impact energy” becomes a scale for judging whether a chemical reaction can be induced in energetic jets and the degree of reflection. Walker and Wasley [14] proposed a method for calculating the shockwave energy, as shown in Equation (4):

(4) $$E=PU_{P}t$$

(5) $$t=\frac{2h}{U_{S2}}$$

(6) $$U_{P}=\frac{-(\rho_{2}C_{2}+\rho_{1}C_{1}+2\rho_{1}S_{1}V)\pm {\left({\left(\rho_{2}C_{2}+\rho_{1}C_{1}+2\rho_{1}S_{1}V\right)}^{2}+4\rho_{1}(\rho_{2}S_{2}-\rho_{1}S_{1})(C_{1}V+S_{1}V^{2})\right)}^{1/2}}{2(\rho_{2}S_{2}-\rho_{1}S_{1})}$$

where *P* is the pressure of the jet impact loading, *t* is the pulse loading time, as shown in Equation (5), *h* is the thickness of a limited target, such as a thin steel target, and *U_p_* is the jet particle velocity, which is calculated by Equation (6), according to one-dimensional shock wave theory. Equations (4) and (5) show that the impact energy is related to the thickness of the target plate impacted by the jet, so the impact energy loaded on the jet can be changed by adjusting the thickness of the target plate in this experiment. The values of the impact parameters are shown in Table 3.

**Figure 13:**
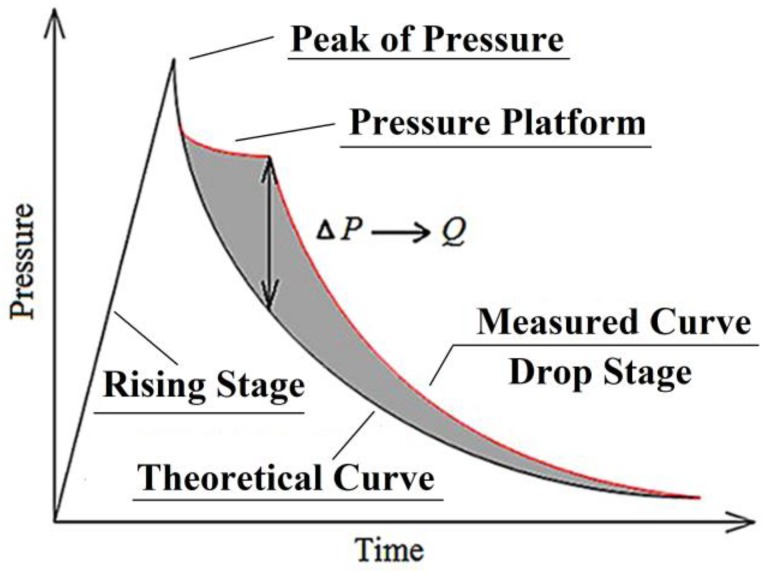
The graphic of the test curve.

Figure 13 shows the graphic of the test curve. The pressure is divided into two stages: the rising stage and the drop stage. After the jet impacts the target plate, the pressure in the container rises sharply during the rising stage, which indicates that the chemical reaction of the Fe/Al energetic jet takes place in this stage. Due to the existence of pressure-relief holes, the pressure gradually decreases, forming the drop stage. There is a pressure platform in the drop stage, which indicates that the self-propagating reaction of the Fe/Al energetic jet occurs continuously at this time, and the energy released is offset by the energy consumed from the pressure-relief hole, which characterizes the continuous reaction of the Fe/Al energetic jet. Therefore, the energy released should consist of two parts: the energy released in the rising stage and the pressure platform. In fact, the pressure detected in the vessel consists of the chemical reaction of energetic substances in the energetic jet and gas expansion caused by the temperature of the jet. Therefore, to study the former, it is necessary to subtract the latter from the total energy, where the pressure caused by the expansion of gas caused by the temperature of the jet is replaced by an inert jet impacting the target plate.

Ames [15] considered the rising stage of pressure in a container to be only a few milliseconds and the *p*-*t* relationship to be basically linear. Therefore, the presence of pressure-relief holes can be neglected in the rising stage of the pressure curve. If the container is closed and the gas mass in the chamber is constant, the relationship between the pressure in the vessel and the release energy during the rising stage of the pressure curve can be calculated as follows [15]:

(7) $$\Delta Q=\frac{V}{\gamma -1}\Delta P$$

where Δ*Q* is the increased energy inside the container, Δ*P* is the increased pressure inside the container, *V* is the volume of container, and *γ* is the ratio of the specific heat of the gas in the container; the chosen value is 1.4. When *P* reaches the peak pressure value, *Q*_1_ is the maximum energy released during the rising stage of the pressure curve. The energy corresponding to the peak pressure is subtracted from the peak pressure energy produced by the inert jet *Q*_Δ_ in the control group, which is the energy released by the chemical reaction of the Fe/Al energetic jet in the rising stage, and quantification of the energy released by the Fe/Al energetic jet is achieved.

To calculate the energy released in the pressure platform, pressure relief should be considered, and Equation (3) cannot be used. Ames [15] considered the relationship between the pressure value and energy value of a non-hermetic container, as shown in Equation (8):

(8) $$\frac{dQ}{dt}=\frac{V}{\gamma -1}\frac{\partial P}{\partial t}+\frac{\gamma PV}{m(\gamma -1)}\frac{dm}{dt}$$

where *m* is the gas mass in the container. Ignoring the viscous and frictional heat conduction of the vessel wall, the flow of the pressure-relief gas can be regarded as one-dimensional steady isentropic flow. An unconstrained pressure-relief container is constructed. There is no heating or heat dissipation flow inside the container, and an ideal gas is used. The upstream and downstream states of the pressure-relief port are set as the standards of subscripts 1 and 2 from the Bernoulli, Equation (9):

(9) $$h_{1}+\frac{v_{1}^{2}}{2}=h_{2}+\frac{v_{2}^{2}}{2}$$

where *h* is the enthalpy, and *v* is the gas flow velocity. The relationship between the enthalpy *h* of a gas in the ideal state and the parameters *P* and *V* is substituted into Equation (9):

(10) $$\frac{v_{2}^{2}}{2}=\frac{v_{1}^{2}}{2}+\frac{\gamma }{\gamma -1}(P_{1}V_{1}-P_{2}V_{2})$$

For the subsonic flow of gas in a chamber, the velocity *v*_1_ of the upstream gas flow is much lower than that of the downstream gas flow; therefore, the *v*_1_ term in Equation (10) can be ignored. The amount of gas per unit time passing through the pressure-relief hole of area *A* at velocity *v*_2_ is shown in Equation (11).

(11) $$\frac{dm}{dt}=\frac{Av_{2}}{V_{2}}$$

By substituting Equation (10) and the equation of state *P* = *ρRT* into Equation (12), Equation (12) can be obtained:

(12) $$\frac{dm}{dt}=\frac{AP_{1}}{T^{1/2}}{\{\frac{2\gamma }{R(\gamma -1)}[{\left(\frac{P_{2}}{P_{1}}\right)}^{2/\gamma }-{\left(\frac{P_{2}}{P_{1}}\right)}^{\frac{\gamma +1}{\gamma }}]\}}^{1/2}$$

where *ρ* is the gas density, *R* is the gas constant and *T* is the gas temperature. When the upstream pressure is equal to or greater than the critical pressure, *v*_2_ = *C*, and Equation (12) can be written as:

(13) $$\frac{dm}{dt}=\frac{AP_{1}}{T^{1/2}}{\{\frac{2\gamma }{R(\gamma -1)}[{\left(\frac{2}{\gamma +1}\right)}^{2/\gamma -1}-{\left(\frac{2}{\gamma +1}\right)}^{\frac{\gamma +1}{\gamma -1}}]\}}^{1/2}$$

The pressure change rate in the vessel can be calculated according to the equation of state, assuming that the air temperature remains unchanged. Substituting $\frac{dn}{dt}=\frac{dm}{Mdt}$ into Equation (13) produces Equation (14):

(14) $$\frac{dP}{dt}=-\frac{RT}{V}\cdot \frac{AP}{M}\sqrt{\frac{\gamma }{TR}}{\left(\frac{2}{\gamma +1}\right)}^{\frac{\gamma +1}{2(\gamma -1)}}$$

Equation (14) describes the theoretical curve of the pressure variation in vessels with pressure-relief holes, but it does not consider the effect of pressure source with continuous release on the pressure in the vessel. Therefore, when the energetic jet impacts the target plate, it releases energy continuously, which slows down the speed of pressure relief and forms the pressure platform in the vessel. The measured pressure value on the pressure–time curve will exceed the theoretical value. By comparing the experimental pressure curve with the theoretical pressure curve, the part of the pressure platform of the former over the latter can be used to represent the energy release of the Fe/Al energetic jet during the process of pressure relief. The difference between the plateau pressure and the corresponding theoretical pressure of each pressure–time curve is substituted into Equations (8) and (14). By solving the above differential equation, the additional release potential value *Q*_2_ in the pressure platform can be obtained. The total release energy *Q*_T_ generated by the chemical reaction of the energetic jet during the impact process can be calculated by Equation (15). A schematic diagram for calculating the release energy of the pressure drop section is shown in Figure 13.

*Q*_T_ = *Q*_1_ + *Q*_2_ − *Q*_Δ_ (15)

where *Q*_T_ is the total energy released by the energetic jet, *Q*_1_ is the maximum energy released during the rising stage of the pressure curve, *Q*_2_ is the additional energy released during pressure platform, and *Q*_Δ_ is the total energy released by the inert jet in the control group.

**Figure 14:**
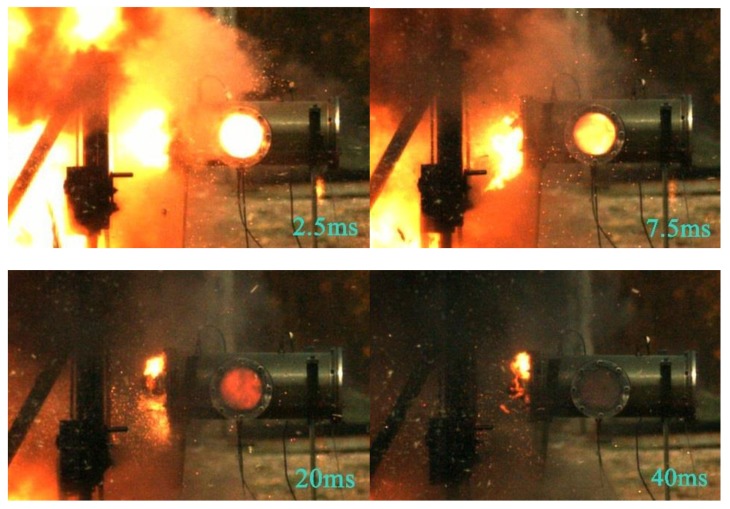
High-speed video of experiment No. 6 with a violent reaction.

**Figure 15:**
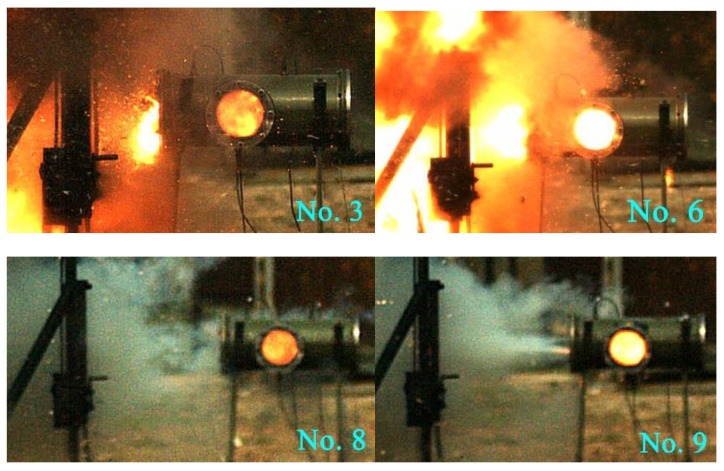
The energy-release phenomenon of the jets at the peak pressure under different schemes.

**Figure 16:**
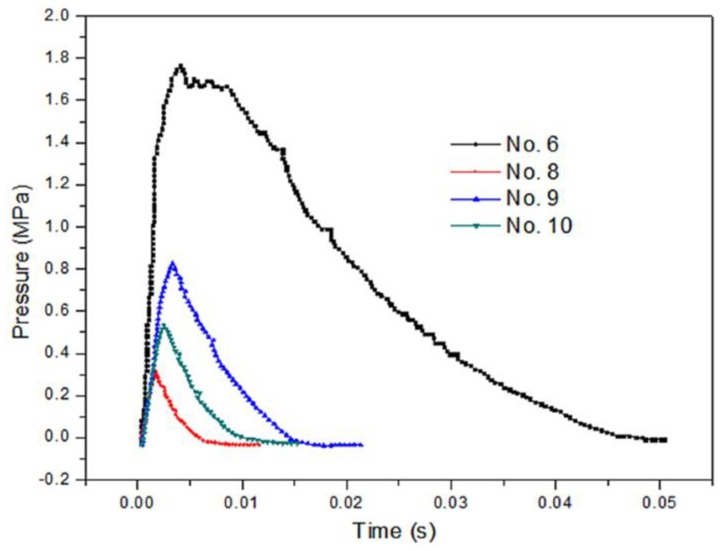
The pressure–time curve of fine-grained Fe/Al energetic jets and control scheme.

**Figure 17:**
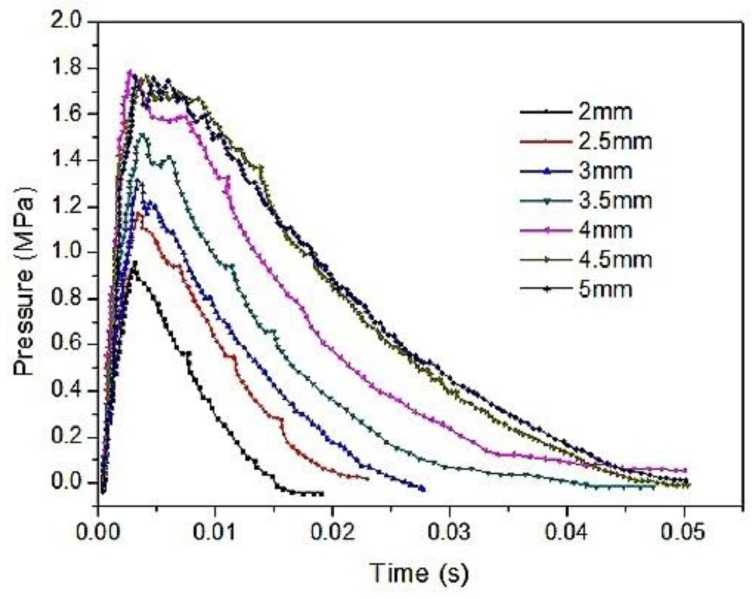
The pressure–time curve of Fe/Al energetic jets impacting the target plate with different thicknesses.

**Figure 18:**
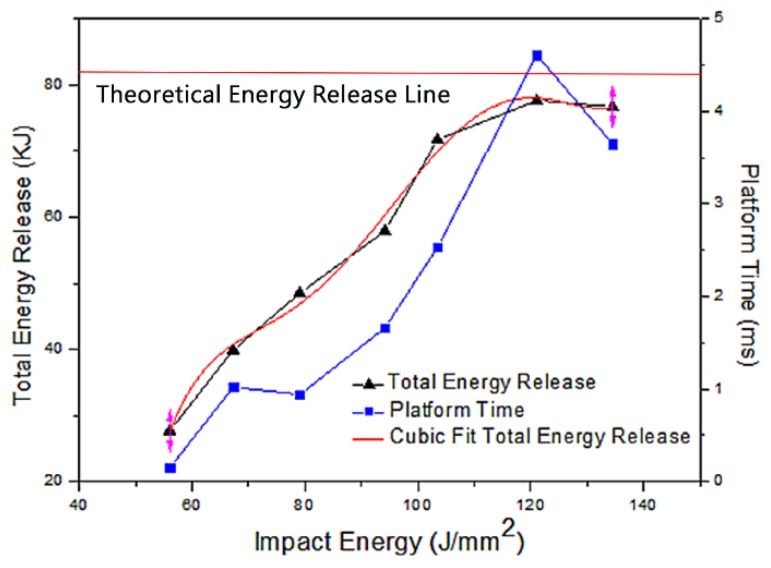
The relationship between the impact energy and energy release and platform time.

**Figure 19:**
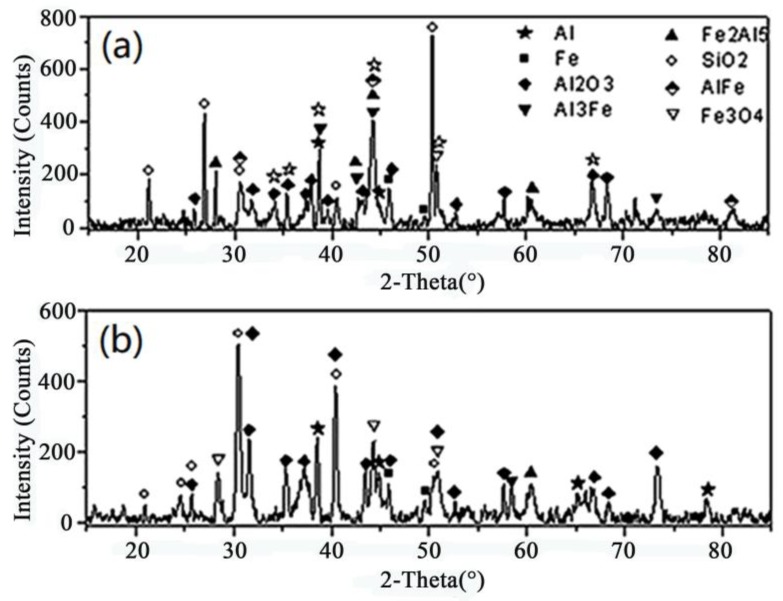
XRD patterns of the reaction products with different thickness targets: (**a**) 2 mm target and (**b**) 5 mm target.

Figure 14 shows a high-speed video of experiment No. 6 with a violent reaction. Figure 15 shows the energy-release phenomenon of the jets at peak pressure under different schemes. Figure 16 shows the pressure–time curve of fine-grained Fe/Al energetic jets and the control scheme. Figure 17 shows the pressure–time curve of Fe/Al energetic jets impacting the target plate with different thicknesses. Figure 18 shows the relation between the impact energy and the energy release and platform time. Figure 19 shows the XRD patterns of the recovered products. Table 4 shows the standard molar enthalpy of formation for the reaction products in the aluminum–iron system. Table 5 compares the overpressure and energy-release values of the different test schemes.

According to Table 4, the theoretical energy-release value of the Fe/Al energetic jet in this experiment can be calculated, so the energy efficiency ratio can be expressed as Equation (16):

*ξ* = *Q*_T_/*Q*_L_ (16)

where *ξ* is the energy efficiency ratio, *Q*_L_ is the theoretical energy-release value, and the calculated value is 81.46 kJ.

From Figure 15, the influence of the impact energy on the energy-release characteristics of the fine-grained Fe/Al energetic jet is obvious. Regarding the discharge of the material from the pressure-relief hole, only a small spark is emitted at a low-impact-energy condition, and a large flame is ejected from the pressure-relief hole at a high-impact-energy condition, indicating that the energetic substances in the jet induce a violent chemical reaction under high-impact-energy conditions. Comparing the impact of an energetic jet and an inert Fe jet on a 5 mm target plate, the flame brightness and amount of the latter are significantly lower than those of the former.

Figure 16 shows that the energy-release effect of the fine-grained Fe/Al energetic jet is much better than that of the inert jet with the same mass. Under the same preparation conditions, the energy release of the fine-grained Fe/Al energetic jet is 9.6 times that of the coarse-grained jet. Under the same particle size, the energy efficiency ratio of the hot-pressed sintered Fe/Al jet is 4.2 times as much as that produced by traditional mechanical processing. The results show that fine particles and proper hot-pressing sintering are beneficial for inducing the maximum energy release of the Fe/Al energetic jet.

Figure 17 and Figure 18 and Table 5 show that as the thickness of the target increases, the impact energy of the jet increases, and the pressure peak, transient energy-release values, and platform time of the fine-grained Fe/Al energetic jet increase. When the target thickness is 4.5 mm and the impact energy is 121.1 J/mm^2^, the total release energy of the Fe/Al energetic jet reaches a peak value of 77.6 kJ, and the platform time reaches a peak of 4.61 ms. When the thickness of the target plate exceeds 4.5 mm, the release energy of the fine-grained Fe/Al energetic jet does not increase further, and the platform time decreases slightly, which indicates that the chemical reaction of the energetic material in the jet is saturated due to the existence of a shock energy threshold at 121.1 J/mm^2^.

Among the schemes, the maximum energy-release efficiency of the Fe/Al energetic jet is 95.3%, which indicates that after reasonable preparation, the Fe/Al jet can fully induce the chemical reaction of energetic substances in the jet after loading a sufficient impact. According to the regular pattern shown in Figure 17, the relationship of the response induced by the shock energy loading of the fine-grained Fe/Al energetic jet can be fit by the function *Q*_T_ = −2167 + 122*I* − 2.7*I*^2^ + 0.029*I*^3^ − 1.5 × 10^−4^ *I*^4^ + 3.1 × 10^−7^ *I*^5^ (*I*: impact energy), as shown in Figure 18.

It can be seen from Figure 19 that, in the low-impact-energy recovered products, the elemental Al content is the highest. Additionally, small amounts of intermetallic composites are found, and no substantial amount of Fe_3_O_4_ is formed, indicating that the aluminum oxidation and aluminum–iron intercalation reactions occur mainly under low-impact-energy conditions. A high-impact-energy condition results in the recovery of only a small amount of metallic elements and composites, most of which are Al_2_O_3_ and Fe_3_O_4_, indicating that a great quantity of oxidative reactions of energetic substances in Fe/Al jet is induced by high-impact energy, as shown in Equations (1) and (2). The results shown in Figure 19 explain the phenomenon of more energy released by a fine-grained Fe/Al energetic jet under high-impact energy, as shown in Figure 18 for the mesoscopic scale.

## 5. Conclusions

This paper describes experimental research and a theoretical analysis of the energy-release characteristics of a fine-grained Fe/Al energetic jet under impact loading. The dynamic mechanical properties of fine-grained Fe/Al energetic materials were studied. An experimental system was established to test the pressure caused by impact loading of a fine-grained Fe/Al energetic jet, and differences in energy release were discussed based on the impact-energy calculation. The following specific conclusions can be drawn:

(1) When the sintering temperature is 550 °C, a solid-state diffusion reaction between Al and Fe begins to take place at a pressure of 10 MPa, forming Fe_2_Al_5_. When the temperature exceeds 600 °C, the diffusion reaction occurs.
(2) Fe/Al materials prepared at different maximum sintering temperatures show the characteristics of brittle materials with poor ductility. The fracture surface reflects intergranular brittle fracturing. When the maximum sintering temperature is 550 °C, the compressive strength of the fine-grained Fe/Al composites is the highest among the different composites. To maintain the activity and strength of the Fe/Al energetic material, the maximum sintering temperature should be 550 °C.
(3) The reaction behavior of the fine-grained Fe/Al energetic jet under impact is related to the impact energy. With increasing impact energy, the energy release of the fine-grained Fe/Al energetic jet increases. The reaction mechanism is as follows: The aluminum oxidation reaction and the aluminum–iron intercalation reaction occur mainly under low-impact-energy conditions, and a great quantity of oxidative reactions of energetic substances is induced by high-impact energy. There is an impact-energy threshold at 121.1 J/mm^2^, at which the chemical reaction of the energetic material in the jet is saturated, and the highest reaction rate is 95.3%.
(4) The Fe/Al energetic jet with fine particles and proper hot-pressing sintering has a higher energy efficiency than those created with coarse-grained and traditional mechanical processing under the same impact conditions.

## Figures and Tables

**Table 1:** Parameters of the Fe/Al composites.

No.	Highest Sintering Temperature (°C)	Density (g/cm^3^)	Mass (g)
1	500	3.59	3.38
2	520	3.65	3.43
3	550	3.74	3.52
4	600	3.88	3.65

**Table 2:** Experimental scheme.

No.	Mass Ratio (Fe:Al)	Target Thick-Ness (mm)	Mass (g)
1	4:6	2	3.84
2	4:6	2.5	3.84
3	4:6	3	3.84
4	4:6	3.5	3.84
5	4:6	4	3.84
6	4:6	4.5	3.84
7	4:6	5	3.84
8	Cu	4	3.84
9	Fe/Al Mix-ture ^a^	4.5	3.88
10	Fe/Al Mix-ture ^b^	4.5	3.84

^a^ The particle sizes of iron and aluminum are the same as those in No. 1, prepared by traditional mechanical processing technology; ^b^ the particle sizes of iron and aluminum are 52 and 104 μm, respectively, and the preparation process is the same as that of No. 1.

**Table 3:** Values of the impact parameters.

*ρ*_1_^a^ kg/m^3^	*ρ*_2_^b^ kg/m^3^	*C*_1_^c^ m/s	*C*_2_^d^ m/s	*S*_1_^e^	*S*_2_^f^	*V*^g^ m/s	*U_p_* m/s	*U_s_*_2__h_ m/s
3660	7850	4624	4569	1.57	1.49	3555	1309	6520

^a^ The density of the jet; ^b^ The density of the target; ^c^ The acoustic velocity of the jet; ^d^ The acoustic velocity of the target; ^e^ The empirical parameters of the jet; ^f^ The empirical parameters of the target; ^g^ The velocity of the jet; ^h^ The shock wave velocities of the target.

**Table 4:** Standard molar enthalpy of formation of the reaction products in the aluminum–iron system (including oxidation).

Product	Fe	Fe/Al	FeA1_2_	FeA1_3_	Fe_2_A1_5_	Al	Al_2_O_3_	Fe_2_O_3_	Fe_3_O_4_
$\Delta H_{f,298}^{\theta }$ (kJ/mol)	/	50.3	79.2	112.3	187.7	/	1669.68	824.2	1118.4

**Table 5:** Comparison of the pressures and energy-release values of the different testing schemes.

No.	Impact Energy (J/mm^2^)	${\bar{P}}_{m}$ (MPa)	Platform Pressure (MPa)	Platform Time (ms)	*Q*_1_ (kJ)	*Q*_2_ (kJ)	*Q*_T_ (kJ)	Energy Efficiency Ratio (%)
1	56.1	0.97	0.88	0.15	35.6	3.5	27.7	34.0
2	67.3	1.17	1.09	1.02	42.9	8.4	39.9	49.0
3	79.1	1.33	1.21	0.94	48.8	11.1	48.5	59.5
4	94.2	1.52	1.39	1.66	55.7	13.6	57.9	71.1
5	103.5	1.77	1.58	2.53	64.9	18.2	71.7	88.0
6	121.1	1.8	1.68	4.61	66.0	23	77.6	95.3
7	134.5	1.78	1.70	3.65	65.3	22.8	76.7	94.1
8	---	0.31	---	---	11.4	---	11.4 ^a^	---
9	---	0.82	---	---	30.1	---	18.7	22.9
10	---	0.53	---	---	19.4	---	8.0	9.9

^a^*Q*_Δ_ takes the release energy of inert jet 11.4 kJ.
